# Thinking about neither death nor poverty affects delay discounting, but episodic foresight does: Three replications of the effects of priming on time preferences

**DOI:** 10.1177/17470218221097047

**Published:** 2022-05-25

**Authors:** Richard J Tunney, Jodie N Raybould

**Affiliations:** School of Psychology, Aston University, Birmingham, UK

**Keywords:** Social priming, impulsivity, scarcity, mortality salience, delay discounting, time preference, replication

## Abstract

We outline three attempts to replicate experiments that reported priming effects on time preferences measured by delay discounting. Experiment 1 tested the claim that images of poverty prime impulsive choice in people from less affluent backgrounds compared with people from more affluent backgrounds. Experiment 2 tested the claim that mortality salience—thinking about death—primes people to place more value on the future than people who thought about dental surgery. Experiment 3 tested the claim that an episodic foresight manipulation primes greater discounting than no episodic foresight. Experiments 1 and 2 failed to replicate the effects of priming on discount rates. Experiment 3 was a successful and very close replication of the effect of episodic foresight on discount rates.

Time preference is a relatively stable individual difference with a large heritable component. It is a predictive factor in a range of life choices and the transition from recreational to addictive behaviours, such as smoking, excessive alcohol consumption, problem gambling, obesity, and financial mismanagement (Odum, 2011). Any intervention that could affect the stability of time preference might, therefore, present the opportunity for improvement to the choices that people make. In recent years there have been several reports that priming can affect time preferences. However, some forms of priming are controversial and may not always be replicable. In three experiments we sought to replicate the effects of priming on a psychophysical measure of time preferences—delay discounting.

## Delay discounting

Delay discounting is a commonly used measure of the time preference component of impulsivity (Rachlin et al., 1991). Delay discounting measures the point at which people are indifferent between a series of hypothetical smaller-sooner and larger-later monetary rewards (e.g., would you prefer £20 now or £100 in 6 months?). The indifference point implies that the subjective value of a delayed outcome is discounted with increasing time to its receipt. This is typically expressed as a parameter called the discount rate that is a measure of an individual’s time preference and is generally considered to be indicative of their general impulsivity. This preference for smaller-sooner rewards over larger-later rewards is analogous to other examples of individual differences in impulsivity that are typically exhibited in behaviour and decision-making. For example, in the well-known Marshmallow Test (Mischel et al., 1989) children are given a choice between one marshmallow immediately, or the opportunity to wait for two marshmallows later. The length of time that a child is willing to wait before choosing the smaller-sooner reward over the larger-later reward seems to be predictive of a wide range of choice behaviours in later life. For example, as adults, children who were less able or willing to delay their gratification tended to have higher body mass indices, be more likely to use illegal recreational drugs, and be more likely to be divorced (Casey et al., 2011).

The association between discount rates and a wide range of personal and public health issues makes time preference a plausible target for psychological intervention. Indeed, similar approaches with other aspects of impulsivity, such as impulse control, have been moderately successful in modifying choice behaviour (Allom et al., 2016). Some claim that choice behaviour such as that measured by delay discounting can be affected by the phenomena of social priming.

## Social priming

Social priming first emerged in the 1980s in response to evidence found in favour of semantic priming. Semantic priming suggests that viewing a word before a comprehension task can lead to faster processing and recognition of related words. For example, being primed with the word “spoon” may lead to faster recognition of the word “fork.” Some researchers argued that a similar effect could be seen in attitudes and behaviour. For example, Srull and Wyer (1979) reported that hostility-related stimuli as a prime made participants more likely to judge ambiguous behaviour in a story as hostile. Similarly, Bargh et al. (1996) reported that people primed with “rudeness” interrupted the researcher more than those primed with “politeness,” and people primed with elderly stereotypes tended to walk more slowly than controls. Since these early studies, many researchers have pursued evidence that social priming affects a surprisingly wide and increasingly bizarre range of phenomena. For example, Vohs et al. (2006) reported that money-related primes reduced prosocial behaviour, Dijksterhuis and van Knippenberg (1998) found primes suggestive of professionalism led to better scores on quizzes, and Lee and Schwarz (2012) found that fishy smells lead to increased suspicion of others. However, many of these studies are difficult to replicate (Chabris et al., 2019; McCarthy et al., 2018; Rohrer et al., 2019; Shanks et al., 2013), casting doubt on the reliability of social priming effects. Consequently, some researchers have published absurd findings to critique social priming, such as Simmons et al. (2011), who found that people gave birth dates nearly a year-and-a-half younger after they had listened to the Beatles’ song “When I’m Sixty-Four” compared with a different Beatles song. This critical commentary on the statistical practices being used by some researchers, and the difficulties in replicating many studies, has led some researchers to almost reject the phenomenon of social priming entirely. Despite this, there is continued research into social priming effects.

At least three studies have reported social priming effects on impulsivity measured using delay discounting. In their study on impulsivity and scarcity, Griskevicius et al. (2013) concluded that adults who reported economic uncertainty during their childhood showed greater discounting after viewing priming images of recession. Their study was based on Life-History Theory in which economic scarcity drives impulsivity and abundance cues self-control (Roff, 2002; Stearns, 1992). In contrast, in their study on mortality, Kelley and Schmeichel (2015) tested Terror Management Theory (Greenberg & Arndt, 2011) and concluded that participants who were asked to think about their own death were significantly more likely to prefer larger rewards later compared with a control group. More recently Bulley et al. (2019) reported an experiment in which discount rates could be affected by merely imagining positive or negative future events. Their study is based on a model of Episodic Foresight as a modifier of behaviour. We report attempts to replicate each of these studies.

## Experiment 1

Does viewing images of economic recession lead to a preference for smaller, but sooner rewards? In their Experiment 1, Griskevicius et al. (2013) showed 168 participant images that either depicted economic recession or natural scenes. After this, participants made 20 choices to indicate their preference for either a small reward the day after the experiment or a larger reward after a delay of 33 days. The smaller-sooner reward values varied between US$9 and US$86. The larger-later reward values varied between US$47 and US$99. Griskevicius et al. also asked participants to complete six questions about their perceived childhood and current economic status. Risk preferences were also measured but we did not seek to replicate this condition.

The results did not show any main effects of prime type or social status. However, there was an interaction between childhood perceived social status and the prime in which the participants who perceived their childhood social status to be relatively impoverished had higher discount rates following the recession prime than similar participants who saw the natural scenes. By contrast, the participants who perceived their childhood social status as relatively affluent had lower discount rates following the recession prime than similar participants who saw the natural scenes.

In the experiment that follows, we sought to replicate this effect using a similar preparation to the one reported in the original study. In addition to the social status items used by Griskevicius et al., we measured delay discounting using the 27-Item Monetary Choice Questionnaire (27-MCQ) (Kirby & Marakovic, 1996). In addition, while the original study was incentive compatible, meaning one of the decisions was real, our study was not incentivised in this way. Our study also uses images of abundance as a control rather than images of nature because we assumed that a greater contrast between conditions would increase the observable effect. Finally, our experiment was conducted online, and we did not attempt to replicate the reported effects on risky decision making.

### Method

#### Participants

We based our sample estimate on large (.89) and medium (.64) effect sizes for high and low socioeconomic status (SES) groups. These values were reported by Rung and Madden (2018) and based on data for a closely related study by Griskevicius and colleagues (Griskevicius et al., 2011). To detect the reported effects, we would require sample sizes of 69 and 130, respectively; however, we opted to use a larger sample size of 241 participants. These participants were recruited remotely through prolific.co in return for £7.50 (US$10.02), and all participants gave written informed consent prior to data collection; 123 participants viewed images of recession and 115 viewed images of abundance; 3 participants withdrew, leaving 238 datasets for analysis (98.76%); 167 participants identified as female, 70 as male, and 1 selected the “other” option. The average age of the sample was 35.77 years (*SD* = 11.51).

#### Procedure

Participants completed a series of short, standardised questionnaires using Qualtrics software. Questions proposed by Griskevicius et al. (2013) measured SES in relation to current circumstances, and then again in reference to childhood circumstances. In our survey greater scores indicated lower social status. Delay discounting was measured using the 27-Item Monetary Choice Questionnaire (27-MCQ) (Kirby & Marakovic, 1996).

### Results

We attempted to conduct our analyses as closely as possible to those reported by Griskevicius et al. In each of our analyses we used the proportion of larger-later choices as our dependent variable. We first performed regression analyses using dummy coded priming images (control versus recession) and the mean centred Childhood and Adult SES scores as predictor variables. The model was significant (*R*^2^ = .076, *F*_3, 234_ = 6.379, *MSE* = .047, *p* < .001). There was no main effect of Prime (*β* = −.079, *SE* = .028, *t* = −1.241, *p* = .216), and no effect of childhood SES (*β* = −.123, *SE* = .003, *t* = −1.846, *p* = .066); however, in contrast to Griskevicius we found a reliable effect of Adult SES (*β* = −.189, *SE* = .003, *t* = −2.864, *p* = .005). Adult SES was negatively correlated with the proportion of larger later choices (*r* = −.232, *p* *<* .001) indicating that less affluent people tended to make more impulsive choices. Despite this, when conducting a simple linear regression without primes we found that Childhood SES was predictive of delay discounting (*R*^2^ = .036, *F*_1, 237_ = 8.839, *MSE* = .049, *p* = .003).

**Figure 1.**
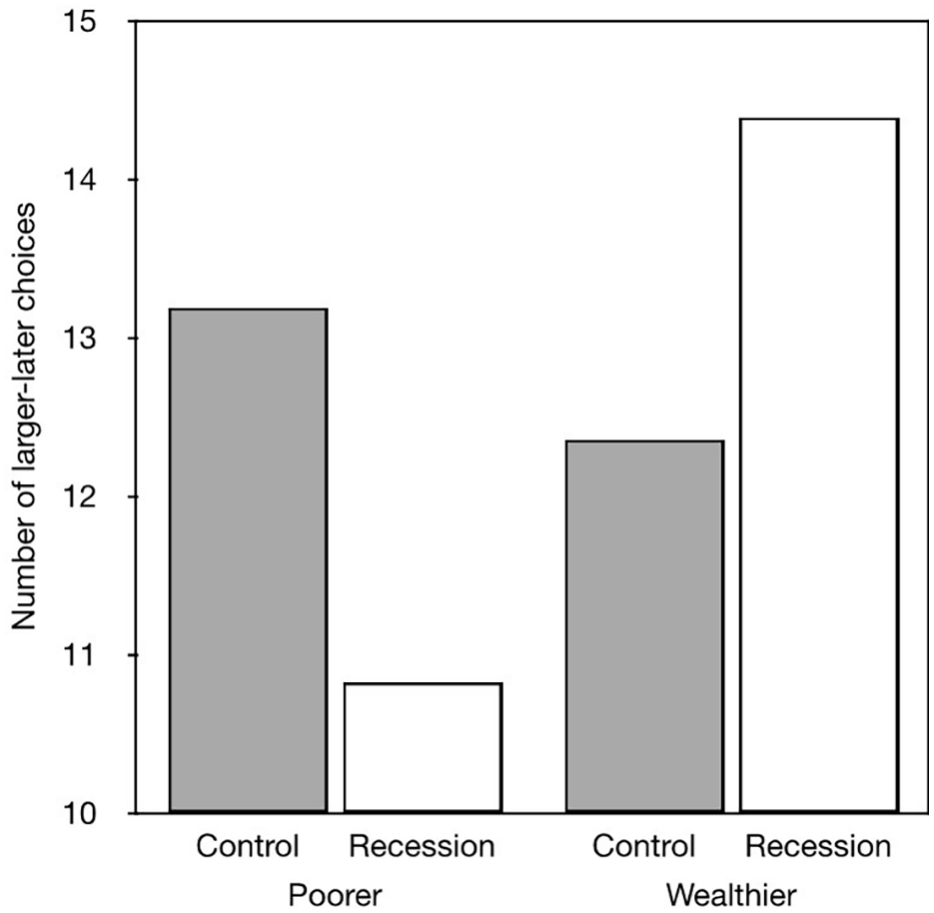
Effects of prime and childhood social status on delay discounting reported by Griskevicius et al. (2013, Experiment 1).

The key finding of interest in the original study is based on an analysis of the priming on subgroups of people who experienced wealthier childhoods compared with people who experienced less affluent childhoods. To define these groups Griskevicius et al. split their sample into groups with childhood SES scores ±1 *SE* from the mean. Griskevicius reported that recession cues primed participants from wealthier childhoods to prefer larger-later rewards, while the same cues primed participants from less affluent childhoods to prefer smaller-sooner rewards (see Figure 1). We repeated this analysis and selected participants whose Childhood SES scores were greater or less than 1 *SD* from the mean score (*M* = 11.47, *SD* = 6.865).

**Figure 2.**
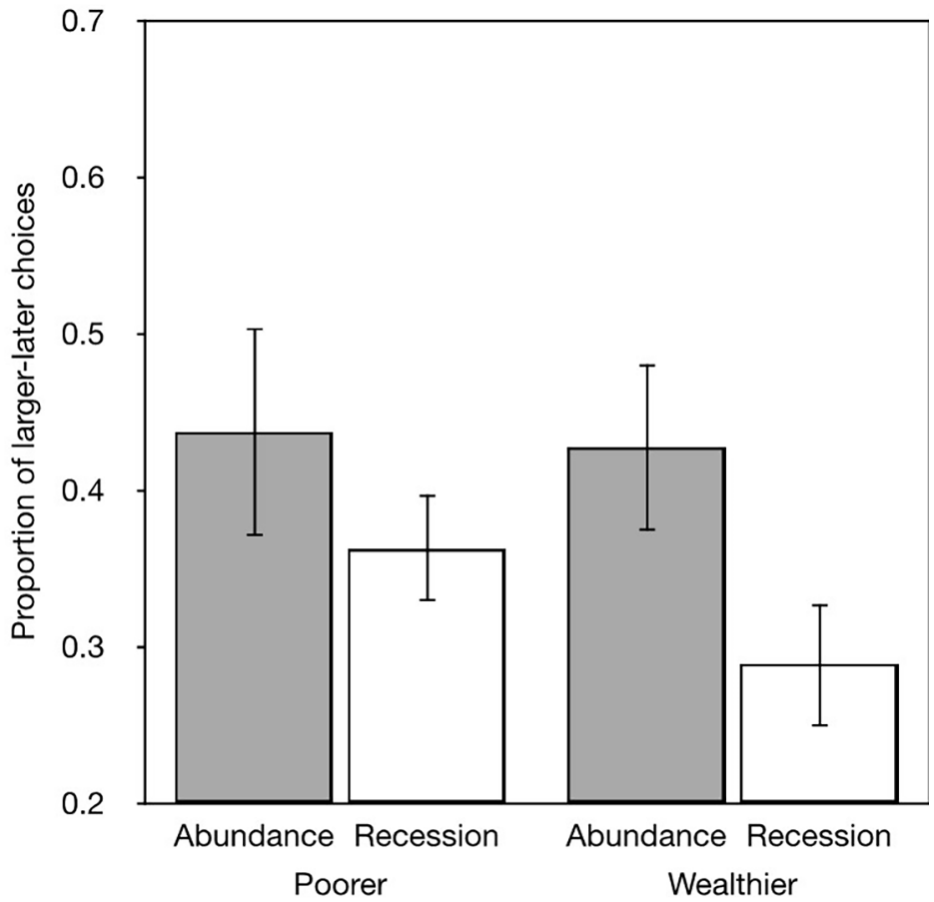
Data from our attempt to replicate Griskevicius et al. (2013; Experiment 1). More and less affluent groups were defined by a split of Childhood subjective social status ±1 *SD* from the mean. Our data do not replicate the claim that either priming affects delay discounting.

In our data (see Figure 2), participants in the more affluent group preferred more larger-later rewards following the Abundance cues (*n* = 15) compared with those who saw the Recession cues (*n* = 23) (*t*_36_ = 2.302, *p* = .027). Participants in the less affluent group showed no such effect, although the trend was for participants who saw the Abundance cues (*n* = 19) to also prefer the larger-later rewards than those who saw the Recession cues (*n* = 5) (*t*_22_ < 1.0). However, this selection criterion resulted in a significant loss of data. Griskevicius et al. do not report their mean and standard deviations, but perhaps our sample has a greater variance. A more inclusive, albeit not strictly proper analysis would be a median split. When we conducted this analysis the participants in the more affluent half tended to prefer the larger-later rewards following Abundance cues (*n* = 61, *M* = .495, *SD* = .250) compared with the Recession cues (*n* = 46, *M* = .415, *SD* = .194) (*t*_105_ = 1.806, *p* = .074). Participants in the less affluent half tended to prefer the larger-later rewards following an Abundance cue (*n* = 55, *M* = .395, *SD* = .189) compared with a Recession cue (*n* = 61, *M* = .376, *SD* = .219), but this did not reach significance (*t*_114_ < 1.0).

### Discussion

We attempted to replicate the effects of priming on delay discounting reported by Griskevicius et al. (2013). In their study, people who reported having relatively impoverished childhoods preferred smaller-sooner rewards after being primed by images of recession, compared with either neutral images or, people who reported having relatively affluent childhoods. We sought to replicate the effect of priming because, as suggested by Odum (2011), delay discounting is a stable individual difference. This would imply that the trait is resistant to external factors and therefore we expected that it would be unaffected by priming. Although, Lempert and Phelps (2016) have reported evidence that intertemporal choice could be malleable and influenced by the context of the choice.

We are also sympathetic to the idea that childhood economic environment might influence adult choice behaviour and so we were interested in replicating this effect as well. However, we did not replicate either effect. In fact, we observed that there was a tendency for people from less affluent backgrounds to prefer larger-later rewards. Does this falsify the life-history hypothesis on which the experiment is based? We suspect not. We think that this may not be a falsifiable proposition because regardless of what pattern of results we obtain, an explanation based on life-history could be proposed. For example, while a preference for smaller-sooner rewards could suggest that childhood scarcity has led to a learnt pattern of over-consumption and therefore immediate need, the opposite result where people from less affluent backgrounds prefer larger-later rewards could indicate that they have become accustomed to hoarding and therefore do not have an immediate need for resources. It is possible to make a directional prediction, but life-history alone may not be sufficient.

It is important to note that our study was not a perfect replication. We used different images of recession, and instead of neutral images of nature, we used images of abundance that we expected might increase the effect. However, we observed no such increase. Similarly, our study was not incentive compatible, although we do not believe that this is a reason for the failure to replicate the original study because we did find an effect of childhood experience on discounting. We also used a standard measure of delay discounting rather than the items used by Griskevicius. This in particular may have impacted the results because Griskevicius used a task where the sooner reward was given the day after the experiment. This is in contrast to the 27-MCQ where the sooner reward is given on that day and is therefore not in the future. This difference between now and not-now choices compared with two not-now choices of different waiting periods could be a vital factor, and in any future replications this would need to be considered. Finally, our experiment was conducted online instead of in the laboratory.

Nonetheless we are confident in our results, in part because the reliable relationship between social status, or rather relative wealth, and individual differences in time preferences is one that we expect from the literature on delay discounting and impulsive choice (Shah et al., 2015; Tunney & James, 2021). In Experiment 2 we attempt to replicate the claim that mortality salience can affect delay discounting.

## Experiment 2

Do people become more impulsive, and seize the day, when they think about their own mortality? Or does thinking about mortality lead people to place a greater emphasis on long-term planning? An experiment reported by Kelley and Schmeichel (2015) suggests that mortality salience can increase future thinking. To elicit mortality salience, participants in a classroom environment were asked to think about and write down the emotions that came to mind when they thought about their own death, and about what they thought would happen to them when they died. The control group was asked to write about a painful dental procedure. Immediately afterwards the researchers gave participants a series of choices between smaller-sooner and larger-later rewards and reported that participants in the mortality salience condition tended to prefer larger-later rewards than the participants in the dental pain condition. That is, thinking about death reduced delay discounting. We preregistered this replication with the Open Science Foundation (osf.io/c5x4z).

### Method

#### Participants

In all, 201 participants were remotely recruited through prolific.co. All participants gave written informed consent prior to data collection. A total of 12 participants failed one or both of the attention checks that we included in the experiment leaving 189 participants for analysis; 92 participants answered a question on mortality and 97 were asked about dental pain; 114 participants identified as male, 72 as female, and 3 as other. The mean age of the sample was 26.75 years (*SD* = 9.704).

#### Procedure

The experiment was conducted online using Qualtrics. Participants were randomly assigned to a Mortality Salience or a Dental Pain condition. We used the same instructions reported by Kelley and Schmeichel (2015) where the Mortality Salience group was asked to

Please briefly describe the emotions that the thought of your own death arouse in you. Enter into the box, as specifically as you can, what you think will happen to you as you physically die and once you are physically dead.

And the Dental Pain control group was asked to “Please briefly try to recall a dental procedure that you have undergone. Enter into the box, as specifically as you can, the emotions that the thought of dental surgery arouse in you.” We required participants to enter at least 300 characters before the experiment would continue. Following this all participants were asked to answer questions on delay discounting, first answering the Kelley and Schmeichel discounting questions, and then the standard 27-MCQ (Kirby & Marakovic, 1996). We included two attention checks recommended by Prolific (n.d.) at the end of each block of choices: “The colour test is simple, when asked for your favourite colour you must click the word purple below. Based on the text you read above, what word have you been asked to click?”

### Results

We first checked that participants engaged with the priming manipulation. There was no significant difference (*t* < 1.0) in the average number of characters recorded by participants in the Mortality Salience condition (*M* = 517.354, *SD* = 314) and Dental Surgery conditions (*M* = 651.421, *SD* = 762.001). We used a free online sentiment analysis tool (https://monkeylearn.com/sentiment-analysis-online/). This categorised responses to the Mortality Salience condition as 61.9% negative and the Dental Surgery condition as 86.5% neutral.

**Figure 3.**
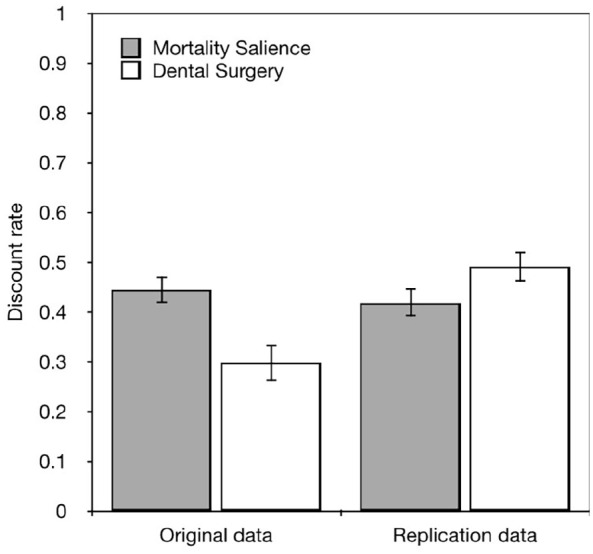
Showing recalculations of the original discount rates reported by Kelley and Schmeichel and the data in our attempt to replicate their effect. The discount rate is calculated using the method described by Weber et al. (2007) and indicates the subjective value of £1 after the delay. Larger values indicate less discounting and smaller values indicate greater discounting. Error bars are standard errors of the means.

Next, we attempted to replicate the analyses reported by Kelley and Schmeichel as closely as possible. We first analysed responses to the Kelley and Schmeichel items using the procedure that they described by finding the value where participants were indifferent between the smaller-sooner rewards and larger-later rewards. Smaller values indicate less discounting. We then calculated the discount rate using the method described by Weber et al. (2007). In this calculation (δ = (*x*^1^/*x*^2^)^1/(t2−t1)^) the amount received that day (*x*^1^ = 50) is divided by the indifference point (*x*^2^), to the power of 1 divided by the start time of “now” (*t*^1^ = 0) minus the future point where money will be received, in 3 months (*t*^2^ = 0.25). A value of 1 indicates no discounting, and smaller values greater discounting. The data are shown in Figure 3. We also recalculated the Kelley and Schmeichel discount rates using the indifference points that were made publicly available because there was an error in the discount rates that they reported. The original Kelley and Schmeichel data reported a discount rate of 1.1 for the indifference point of $105. If we understand the value of 1 to indicate no discounting, then a value above this would suggest less discounting than nothing. In fact, when we inputted this indifference point into the Weber calculation (50/105)^1/ (0.25−0)^ we obtained a discount rate of 0.05, indicating greater discounting for the larger indifference point.

The data were then entered into an independent samples *t*-test. We did not find a significant difference in discount rates between the Mortality Salience and Dental Pain conditions (*t*_187_ = −1.714, *p* = .088). This suggests that thinking about death did not reduce delay discounting. On the contrary, although it was not a significant difference the trend appeared to be in the opposite direction. Our recalculation and reanalysis of the discount rates reported by Kelley and Schmeichel does show a significant difference (*t*_116_ = −3.126, *p* = .002) and a large effect size (Cohen’s *d* = .5767). An a priori power analysis suggests that a sample of 160 is required to detect this effect at 1 − b. Our sample of 189 participants was therefore sufficient to detect this effect but we were unable to replicate it.

We next examined discount rates by estimating the logarithm of the hyperbolic discount function (log-*k*) from the standard 27-MCQ discounting items. These data confirmed that thinking about death (*M* = −2.288, *SD* = .654) does not change discount rates compared with thinking about dental surgery (*M* = −2.349, *SD* = .689) (*t*_187_ < 1.0). To test the validity of the items that Kelley and Schmeichel used to measure discounting we compared means with the 27-MCQ. The two measures did correlate with one another (*r*_182_ = −.515, *p* < .001) suggesting that the Kelley and Schmeichel questions are likely measuring discounting as intended.

### Discussion

We attempted to replicate the effect of mortality salience on delay discounting reported by Kelley and Schmeichel (2015). Despite having a sufficient sample size, and a very close method including using the same items, we were unable to replicate their effect. One potential explanation for this is that the effects of mortality salience on discounting may be smaller than originally suggested by Kelley and Schmeichel, and therefore a larger sample size would be required to find significant results. Finally, in our third experiment we attempt to replicate a more recently reported example of priming time preferences.

## Experiment 3

Can our imagination of future events effect preferences for smaller-sooner or larger-later rewards? Recently, Bulley et al. (2019) reported an experiment in which discount rates could be affected by merely imagining positive or negative future events. Episodic foresight, or the act of thinking about future events, has been shown to affect discount rates in a number of studies (e.g., Bulley & Gullo, 2017; Peters & Büchel, 2010), including a meta-analysis by Rösch et al. (2021) that suggested episodic future thinking has a statistically significant and medium effect on monetary and health-related intertemporal choice. It has also been shown to influence hypothetical decisions about alcohol consumption (Bulley & Gullo, 2017). In this experiment we sought to replicate the results reported by Bulley et al. (2019). Their study was preregistered, and the scientific reporting was of a sufficiently high standard that we were able to perform a near perfect replication of their method.

### Method

#### Participants

We recruited 300 participants using prolific.co in return for £2.50. There were 301 participants in the original study, with 297 remaining after exclusions (Bulley et al., 2019). All participants gave written informed consent prior to data collection. The average age was 26.44 years (*SD* = 7.173); 183 participants were female, 112 were male, 3 were either non-binary or third gender, and 1 preferred not to indicate their gender.

#### Design and procedure

We followed the description of the procedure reported by Bulley et al. as closely as possible. First, the participants completed a visual analogue mood scale (*Answer the following question by selecting the value from 0 to 10 which most accurately represents your mood from* 0 = *Happy to* 10 = *Sad*). This scale is a reversed version of the Bulley et al. scale, but this change is unlikely to affect results. Next, the participants were randomly assigned to one of three conditions that differed according to the items shown in Table 1. In each condition the participants were shown positive, negative, or neutral events and asked to select five that they considered to be the most relevant to them. The five items that they selected were then used to cue episodic foresight during the delay discounting part of the procedure. The instructions were as follows.

Before each question you will be asked to imagine yourself in a particular scenario. For each prompt you should take a moment to imagine yourself experiencing the event as vividly as possible. Produce detailed images of the events being imagined and concentrate on those images attentively. Include as much emotional and background detail as you can [e.g., where are you, what do you do, who is with you, what does it look and sound like, how does it make you feel?]. You will then be asked to choose between different amounts of money over different time periods. The choices are purely hypothetical, and you will not receive the money in the choices, but we want to understand how long you would be willing to wait for different amounts of money. Please answer the question without relating your decision to the event. Just picture the event actually happening before making your choice.

On each trial participants were shown one of the five scenarios that they selected and one of 27 choices between an immediate monetary reward and a larger delayed monetary reward based on the 27-MCQ. This was followed by a standard attention check. The participants were then asked to indicate how vividly they were able to imagine the scenarios, how personally relevant the scenarios were to them, and how strong their emotions were when imagining the scenarios (each on a scale from 1 = *not at all*, to 7 = *very*). Finally, the participants completed the Barratt Impulsiveness Scale (BIS-brief) (Steinberg et al., 2013), Penn-State Worry Questionnaire (PSWQ) (Meyer et al., 1990), and the Patient Health Questionnaire depression inventory (PHQ-9) (Kroenke et al., 2001). The original authors had no specific hypothesis about these scales, and nor did we. However, they did hypothesise that positively valenced episodic foresight would result in reduced delay discounting compared with a neutral control, while the negatively valenced prime would lead to increased delay discounting compared with controls. We did not include the Balloon Analogue Risk Task because there were no effects of the episodic foresight manipulation in the original study.

**Table 1.** Conditions and stimuli used to prime episodic foresight in Experiment 3.

Episodic foresight		
Positive	Negative	Neutral
Dinner party	Getting sick	Using a pencil
Visiting loved ones	Traffic accident	Leaning on a table
Going on holiday	Hurt by animal	Using a bowl
Birthday party	Injury after falling	Entering a building
Seeing live music	Getting an infection	Opening a cabinet
Success at university	Assault by stranger	Sitting on a chair
Going to the beach	Food poisoning	Picking up some scissors
Hanging out with friends	Seeing an intruder	Holding a hammer
Winning an award	Burn on hand	Opening curtains
Spending time in nature	Venomous bite	Folding up paper

### Results

In their study, Bulley et al. reported that participants did not rate the vividness of imagery significantly differently across groups. However, the positive group rated imagery as significantly more personally relevant while invoking more positive feelings than the neutral group, and the negative group rated imagery as significantly less relevant while invoking more negative feelings than the neutral group. Despite this, controlling for the difference in later analyses did not affect their results. The participants in our study reported significantly more personal relevance (*t* = 3.218, *p* = .002), and valence (*t* = 3.958, *p* < .001) in the positive group compared with the neutral group. However, the difference in means between vividness ratings was not significant (*t* = .662, *p* = .509). In comparison, the negative group ratings were not significantly different from neutral ratings for vividness (*t* = 1.382, *p* = .169), valence (*t* = 1.881, *p* = .062), or personal relevance (*t* = .064, *p* = .949). Table 2 shows the mean values found for each group. It is important to note here that the participants in our study were asked to rate the strength of emotion rather than rate emotional response as positive, negative, or neutral. However, as with Bulley et al.’s study, controlling for the differences in latter analyses did not affect the results and so this is unlikely to have affected the replication.

**Table 2.** Descriptive statistics for event cue ratings in each condition of the original study and the replication.

		Original study		Replication	
		*M*	*SE*	*M*	*SE*
Neutral	Vividness	5.378	0.092	4.908	0.148
	Valence	4.483	0.058	4.031	0.161
	Personal relevance	5.103	0.116	4.735	0.166
Positive	Vividness	5.376	0.085	5.040	0.133
	Valence	5.881	0.068	4.901	0.150
	Personal relevance	5.624	0.073	5.446	0.146
Negative	Vividness	5.161	0.091	4.620	0.147
	Valence	2.369	0.071	4.460	0.162
	Personal relevance	4.647	0.097	4.720	0.158

*SE*: standard error.

We used the same regression model reported in the original study with log-*k* as the dependent variable, and age, gender (dummy coded as male vs. female and others), current mood, and condition (dummy coded as Positive vs. Neutral, and Negative vs. Neutral) as predictor variables. The model was significant (*R*^2^ = .057, *F*_5, 298_ = 4.577, *MSE* = .678, *p* < .001). The coefficients for the predictor variables are shown in Table 3. Condition was a reliable predictor of discount rates for both the Positive and Negative conditions relative to the Neutral conditions after controlling for age, mood, and gender, none of which were reliable predictors. For completeness, we ran the same analyses on the data from the original study excluding the same four participants excluded from the original analysis. The results of our analysis of the original data are identical to the published report (*R*^2^ = .088, *F*_5, 300_ = 1.892, *MSE* = .338, *p* < .001).

**Table 3.** Coefficients for the predictor variables in regression analysis of a log-k transformation of 27-MCQ data for the original study and the replication.

Predictor	Original study					Replication				
	*b*	*SE*	β	*t*	*p*	*b*	*SE*	β	*t*	*p*
Intercept	−1.794	0.234		−7.683	<.001	−2.011	0.225		−8.957	<.001
Age	0.000	0.008	−0.003	−.047	.962	0.008	.007	0.065	1.147	.252
Gender: male vs female and other	0.142	0.072	0.110	1.963	.051	0.045	0.099	0.026	0.457	.648
Baseline mood	−0.034	0.021	−0.090	−1.605	.110	0.002	0.021	0.007	0.121	.904
Condition: Positive vs neutral	−0.355	0.082	−0.287	−4.319	<.001	−0.451	0.118	−0.252	−3.826	<.001
Condition: Negative vs neutral	−0.288	0.083	−0.224	−3.487	<.001	−0.492	0.118	−0.274	−4.169	<.001

*SE*: standard error.

#### The proportion of larger-later choices

**Figure 4.**
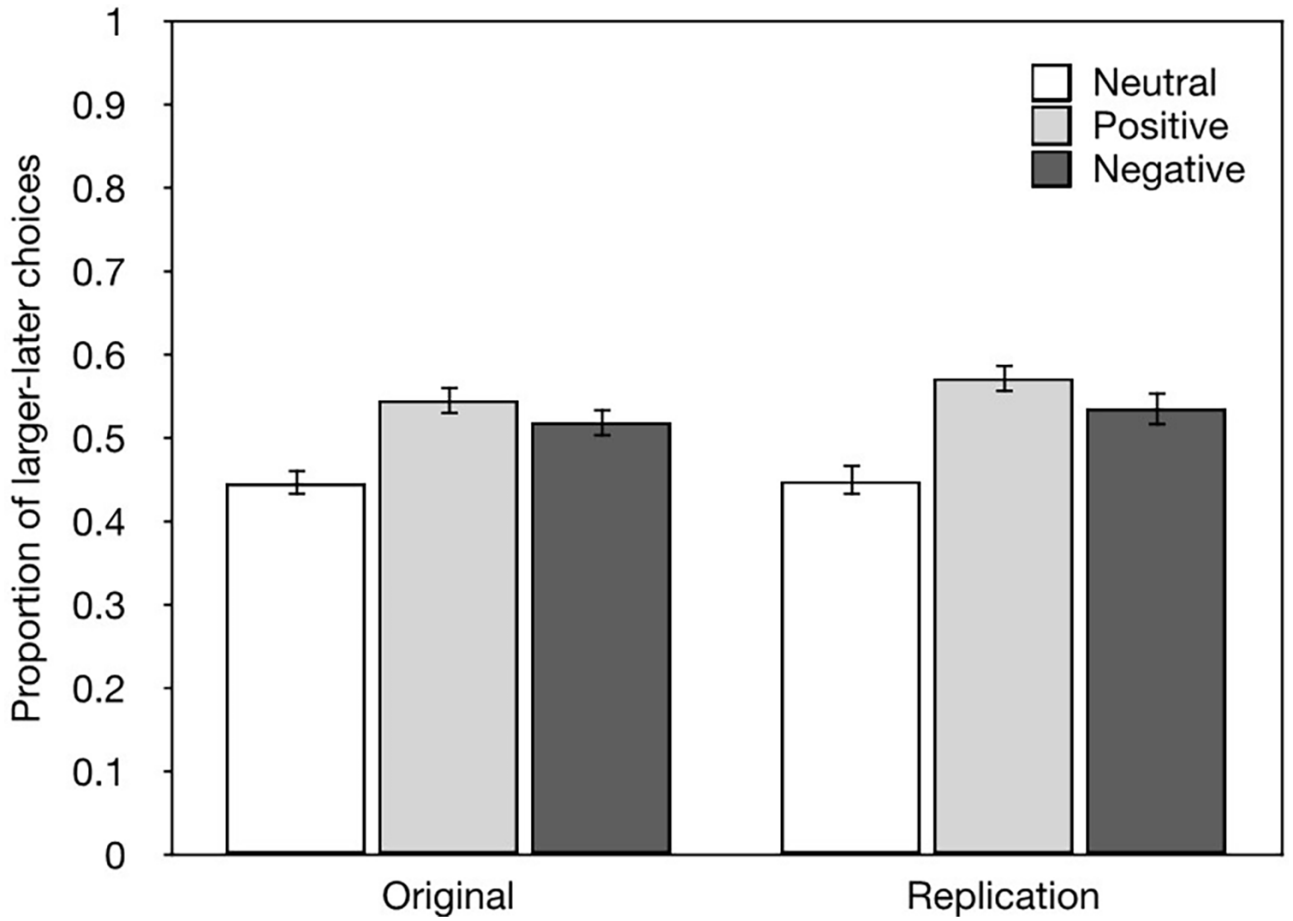
Showing the proportion of larger-later choices by condition for this replication and the original study.

The proportion of larger-later choices is the basic untransformed measure of time preference. These data are shown in Figure 4 for this replication and the original study. We ran a one-way ANOVA on the proportion of larger-later choices in our data. This revealed a main effect of Condition (*F_2,299_* = 10.945, *MSE* = .035, *p* < .001). Simple *t*-tests showed that the proportion of larger-later choices was greater in the Negative condition than in the Neutral condition (*t*_196_ = −3.094, *p* = .001), and also greater in the Positive condition than in the Neutral condition (*t*_198_ = −4.781, *p* = .001). Next, we repeated the same analysis on the data reported in the original study. This revealed a main effect of Condition (*F_2,297_* = 9.992, *MSE* = .026, *p* < .001). Simple *t*-tests again showed that the proportion of larger-later choices was greater in the Negative condition than in the Neutral condition (*t*_196_ = −3.284, *p* = .001), and also greater in the Positive condition than in the Neutral condition (*t*_198_ = −4.364, *p* = .001).

### Discussion

We attempted to replicate the effect of episodic foresight on delay discounting reported by Bulley et al. (2019). In their study they reported that the proportion of larger-later choices was greater in both the Negative and Positive conditions compared with the Neutral condition. In contrast to the previous two experiments, we were able to replicate this result very closely. All three experiments were conducted online, and we see no reason to attribute the failure to replicate two out of three experiments to this feature of our method. Bulley et al. did, however, preregister their analysis and the scientific reportage was of a very high standard that permitted us to copy their methods very closely.

In addition, participants in both the Bulley et al. study and our replication were asked to select their own priming scenarios. This likely led to stronger emotional manipulation due to the increased relevance of the primes. Priming also took place before every individual decision as opposed to at the beginning of the experiment. In Experiments 1 and 2 the effect of the singular case of priming likely led to reduced effects over time. Together these factors may have played a part in the successful replication of the effect that was reported in Experiment 3, but not of the effects in Experiments 1 and 2. Nonetheless, we were able to replicate some of the effects that might have been expected had the prime manipulations worked in the first two experiments.

## General discussion

We report attempts to replicate three separate studies of priming effects on time preferences. In their study on the effects of childhood scarcity on adult impulsivity, Griskevicius et al. (2013) found that images of recession primed participants who reported a less affluent childhood experience to prefer smaller-sooner rewards on a delay discounting task, compared with participants who reported more affluent childhoods. Kelley and Schmeichel (2015) reported that participants who were asked to think about their own mortality were primed to prefer larger-later rewards in comparison to those who thought about dental pain. In trying to replicate these results we found that priming had no significant effect on delay discounting scores in either case, raising a question mark over the reliability of the original reports. Bulley et al. (2019) reported an experiment in which discount rates were affected by merely imagining positive or negative future events. We were able to replicate this experiment. In fact, our results very closely match those reported in the original study.

Our motivation to conduct this work was based on a genuine interest in time preferences as a relatively stable individual difference that is associated with poor life outcomes and addictive behaviours. Although, like many researchers we have followed the replication crisis in psychology with interest, it is not our intention to add fuel to that debate. Instead, we seek an understanding of why some people might be more impulsive than others, and to try and determine whether or not impulsive choice can be modified. These are questions that have significant implications for individuals and wider society.

Our findings raise the interesting question of why two experiments failed to replicate but one successfully replicated. The effect of early childhood experience reported by Griskevicius et al. is a very plausible source of individual differences in time preferences. There is abundant evidence that impulsivity has a large environmental component in its heritability (Bezdjian et al., 2011) and evidence from behavioural ecology suggest that scarcity is a plausible mechanism that drives impulsivity in other species (Andrews et al., 2015). Similarly, mortality salience is a plausible motivating factor in decisions that have long time horizons, although one could develop an argument consistent with either an increase or a decrease in discount rates. For example, if mortality salience led to an increase in discount rates, and so an increase in impulsivity, we could explain this as a growing awareness that we may not actually see the delayed rewards and it would be more sensible to take the smaller-sooner outcomes. On the contrary, if mortality salience reduced impulsivity, one might conclude that this is because thinking about death causes us to place greater value on the future. It is this latter conclusion that was reached by Kelley and Schmeichel (2015). Bulley et al. hypothesised that positively valenced episodic foresight would result in reduced delay discounting compared with a neutral control, whereas negatively valenced episodic foresight would lead to increased delay discounting compared with controls. However, in their experiment, and our replication of it, thinking about future events led to reduced discount rates for both positive and negative future events. We remain open minded about why we were able to replicate the effects reported by Bulley et al., but not by Griskevicius et al. or Kelley and Schmeichel. One possibility is that their manipulations were much more specific while the episodic foresight manipulation elicits more general thinking about future events.

Interestingly, our findings support the meta-analysis reported by Rung and Madden (2018). They examined whether different manipulations could effectively reduce discounting, including priming and episodic future thinking. They found that episodic future thinking produced large and reliable reductions in discounting, with little study variability. In comparison, priming only produced modest reductions with moderate study heterogeneity.

Despite the continued lack of replicable findings in studies like these, some researchers have suggested that social priming studies still have a place in research. By using more rigorous statistical methods to find smaller but more valid findings researchers like Papies (2016) have suggested that pre-existing interest in the priming topic is important. They found that people who want to be thinner will make better food choices when primed with words like “diet” and “thin.” Similarly, Lodder et al. (2019) looked at several priming studies and concluded that significant but small effects were present when the priming related to a goal the participants cared about. This might somewhat help to explain the replicability of Bulley et al., because participants selected priming scenarios that were most relevant to them.

In addition to exploring social priming, we hypothesised that scarcity during childhood could contribute to the development of time preference impulsivity in adulthood regardless of a priming effect. We found a significant relationship between the Griskevicius childhood measure of SES and delay discounting, supporting this hypothesis. Previous studies into how childhood experiences can influence the development of impulsivity have found that adverse childhood experiences can lead to higher impulsivity in adulthood. For example, childhood exposure to multiple adverse experiences is related to poor self-control (Shin et al., 2018). Similarly, Brodsky et al. (2001) reported significantly higher impulsivity scores in participants who had experienced childhood abuse, and Roy (2005) found a small but significant relationship between childhood trauma and impulsivity measured by the Barratt Impulsivity Scale. In further exploring this relationship, Oshri et al. (2019) found that adults who reported more childhood mistreatment performed worse, and displayed lower neural response, during a difficult working memory task. They reported that neural activity significantly mediated the relationship between childhood mistreatment and trait impulsivity and suggested that changes in neurocognitive functioning may underpin the relationship between mistreatment and trait impulsivity. This could suggest that childhood scarcity would need to be significant enough to cause a change in neurocognitive functioning before it has an impact on adult impulsivity, which might help explain the relatively small effect size found in our study.

Alternatively, it may be that the priming effects that we were not able to replicate are simply not sufficiently robust to be replicated. We note that previous high profile reports of priming have failed to replicate in both general tasks (Sherman & Rivers, 2021) and specifically in financial decision-making (Shanks et al., 2013). Nonetheless, we were able to very closely replicate the methods and results reported by Bulley et al. We are optimistic that episodic foresight could indeed play an important role in future research on time preferences and their role in impulsive behaviours, and in the development of future interventions to reduce harmful impulsive choice.

## Supplemental Material

sj-xlsx-1-qjp-10.1177_17470218221097047 – Supplemental material for Thinking about neither death nor poverty affects delay discounting, but episodic foresight does: Three replications of the effects of priming on time preferences Click here for additional data file. Supplemental material, sj-xlsx-1-qjp-10.1177_17470218221097047 for Thinking about neither death nor poverty affects delay discounting, but episodic foresight does: Three replications of the effects of priming on time preferences by Richard J Tunney and Jodie N Raybould in Quarterly Journal of Experimental Psychology

sj-xlsx-2-qjp-10.1177_17470218221097047 – Supplemental material for Thinking about neither death nor poverty affects delay discounting, but episodic foresight does: Three replications of the effects of priming on time preferences Click here for additional data file. Supplemental material, sj-xlsx-2-qjp-10.1177_17470218221097047 for Thinking about neither death nor poverty affects delay discounting, but episodic foresight does: Three replications of the effects of priming on time preferences by Richard J Tunney and Jodie N Raybould in Quarterly Journal of Experimental Psychology

sj-xlsx-3-qjp-10.1177_17470218221097047 – Supplemental material for Thinking about neither death nor poverty affects delay discounting, but episodic foresight does: Three replications of the effects of priming on time preferences Click here for additional data file. Supplemental material, sj-xlsx-3-qjp-10.1177_17470218221097047 for Thinking about neither death nor poverty affects delay discounting, but episodic foresight does: Three replications of the effects of priming on time preferences by Richard J Tunney and Jodie N Raybould in Quarterly Journal of Experimental Psychology
